# Keeping up with the COVID's—Could siRNA‐based antivirals be a part of the answer?

**DOI:** 10.1002/EXP.20220012

**Published:** 2022-07-14

**Authors:** Helen Forgham, Aleksandr Kakinen, Ruirui Qiao, Thomas P. Davis

**Affiliations:** ^1^ Australian Institute for Bioengineering and Nanotechnology The University of Queensland Brisbane Queensland Australia; ^2^ Institute of Biotechnology, HiLIFE University of Helsinki Helsinki Finland

**Keywords:** antivirals, COVID‐19, SARS‐CoV‐2, siRNA, vaccines, virus

## Abstract

Coronavirus disease 2019 (COVID‐19) is a highly contagious viral disease caused by severe acute respiratory syndrome coronavirus 2 (SARS‐CoV‐2). This deadly infection has resulted in more than 5.2 million deaths worldwide. The global rollout of COVID‐19 vaccines has without doubt saved countless lives by reducing the severity of symptoms for patients. However, as the virus continues to evolve, there is a risk that the vaccines and antiviral designed to target the infection will no longer be therapeutically viable. Furthermore, there remain fears over both the short and long‐term side effects of repeat exposure to currently available vaccines. In this review, we discuss the pros and cons of the vaccine rollout and promote the idea of a COVID medicinal toolbox made up of different antiviral treatment modalities, and present some of the latest therapeutic strategies that are being explored in this respect to try to combat the COVID‐19 virus and other COVID viruses that are predicted to follow. Lastly, we review current literature on the use of siRNA therapeutics as a way to remain adaptable and in tune with the ever‐evolving mutation rate of the COVID‐19 virus.

## Graphical abstract

COVID‐19 is a highly contagious viral disease that has claimed the lives of millions of people worldwide. The virus continues to evolve, and each time it does, there is a greater risk that the vaccines and antivirals initially developed to target this deadly infection will no longer be therapeutically viable. Here we discuss the vaccines and the latest antiviral treatment strategies that are under investigation. In particular, we discuss siRNA and why it could be an important piece of the puzzle in the ongoing fight against COVID‐19.

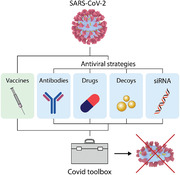

## 1 INTRODUCTION

The first reports of a new idiopathic, severe respiratory infection were announced in the media in December 2019. Initially, all cases were reported to be occurring in only one country.^[^ ^1^ ^]^ But this was just the start. The viral infection now known as COVID‐19 swept quickly throughout the world, causing an escalating global pandemic by March 2020. According to the World Health Organisation's COVID‐19 weekly epidemiological update, COVID‐19 has caused more than 5.2 million deaths and despite our best attempts at vaccinating people against it, the attack by the COVID‐19 virus appears relentless, with some countries going through their ‘fourth wave’ of pandemic with a virus group that now includes the highly transmissible omicron (B.1.1.529) variant.^[^ ^2^ ^]^ To combat COVID‐19 and the other deadly COVID's that are predicted to follow, different treatment strategies should be investigated that work uniquely to target the viruses because as we will demonstrate here, each therapeutic class presents with its own strengths and weaknesses when it comes to treating the virus and its variants. This multimodal approach will provide a toolbox of diverse therapeutics that may be the only way that we are guaranteed to ‘keep up’ with the mutational prowess of these highly virulent pathogens and therefore, there is an urgent need for the discovery and implementation of a COVID medicinal toolbox. This review looks at the various methods being adopted in this respect, and as COVID‐19 continues to dominate our lives, discusses whether short interference RNA (siRNA) should be added to the toolbox, where it could potentially be used as a non‐immunogenic antidote through silencing virulent genes with precision and high specificity.

### 1.1 COVID‐19 transmission and diagnosis

Coronavirus disease 2019 (COVID‐19), is a highly contagious viral disease caused by severe acute respiratory syndrome coronavirus 2 (SARS‐CoV‐2).^[^ ^3^ ^]^ When COVID‐19 was first investigated, initial epidemiological studies found that males over 51 years of age were most at risk of contracting COVID‐19. However, the more recent identification of the Omicron variant in South Africa and its prevalence in infecting young people has made it clear that this is a virus that does not discriminate, although patient outcome is still highly correlated with increased age.^[^ ^4^ ^]^ Transmission rates are greatest in places where there is prolonged contact, which is why large indoor events, households and care facilities are breeding grounds for the virus.^[^ ^5^ ^]^ COVID‐19 is an RNA virus of the Coronaviridae family and fits within the Betacoronavirus subgroup, classified as able to infect a human host.^[^ ^4^, ^6^ ^]^ It is one of seven identified Coronaviridae viruses in the beta subgroup, and although most are moderate in their level of severity, this group also contains previously life‐threatening viruses, middle east respiratory syndrome (MERS) and SARS.^[^ ^7^ ^]^ Characteristically, the viral particles (virions) of the Coronaviridae beta group ingress through the nose, the virus hijacking epithelial cells in the nasal cavity.^[^ ^8^ ^]^ As the virus begins to grow in number, the virions pass down through the respiratory tract and in instances of mild to critical COVID‐19 infection, travel into the lungs.^[^ ^8^ ^]^ It, therefore, makes sense that the most common symptoms of COVID‐19 are cough and pneumonia.^[^ ^9^ ^]^ Transmission to a host mostly occurs when viral particles are relayed from a positive donor through droplets and aerosols that are subsequently inhaled by a healthy recipient.^[^ ^10^ ^]^ However, the infection can also come about from direct contact with an infected person, or from a surface that an infected person has touched.^[^ ^10a^ ^]^ Additionally, because COVID‐19 is a zoonotic virus, thought initially to be transferred from bats to humans, contact with infected animals is a major route of transmission.^[^ ^11^ ^]^ The presence of COVID‐19 has been identified in human stool samples, blood, eye secretions and semen, although the transmissibility from these biological samples remains to be fully investigated.^[^ ^12^ ^]^ Early physical symptoms vary significantly between patients and may also be linked to the severity of infection (Table 1).^[^ ^13^ ^]^ Approximately 80% of people infected will be asymptomatic or not go beyond an upper respiratory infection.^[^ ^14^ ^]^ The remaining 20% routinely experience peripheral and subpleural lung infiltration and when this happens, COVID‐19 can become life‐threatening.^[^ ^15^ ^]^

**TABLE 1:** COVID‐19 physical symptoms and pathological escalation

Physical symptoms of COVID‐19 infection		
Fever, dry cough, lethargy, shortness of breath, headache, muscle pain, sore throat, nasal congestion, chest pain, diarrhea, nausea, vomiting, chills, sputum production, loss of taste and smell		
COVID‐19 escalation	Mild	No *ICU placement required: Fever, cough, fatigue, ground‐glass opacities, presence of mild pneumonia
	Severe	Patient requires *ICU placement: Laboured breathing, blood oxygen saturation ≤93%, respiratory frequency ≥30/min, partial pressure of arterial oxygen to fraction of inspired oxygen ratio < 300. Partial lung infiltration within 24 to 48 h
	Critical	Patient requires *ICU placement: Onset of acute respiratory distress syndrome (ARDS), respiratory failure, septic shock, multiple organ failure

A positive COVID‐19 diagnosis comes from isolation of the virus, followed by real‐time polymerase chain reaction (RT‐PCR) identification of COVID‐19 specific viral markers including RdRp (RNA‐dependent RNA polymerase), E (virus envelope) and N (virus nucleocapsid) genes, and ORF1ab gene (open reading frame 1a and 1b) that are found in the sputum and saliva samples of a positive person.^[^ ^16^ ^]^ The incubation period of COVID‐19 was first predicted to be ∼5 days. Reports suggest that his number dropped to ∼4 days with the delta variant and then to ∼3 days with the omicron variant.^[^ ^17^ ^]^ During this time, the capability of transmission from an infected individual to a healthy recipient (basic reproductive number) is suggested to be between 2.24 and 3.58.^[^ ^18^ ^]^

Unfortunately, the effects of the COVID‐19 virus are not limited to the symptoms occurring during the initial infection. The long‐term consequences (sequela) of COVID‐19 can continue for months to come. For example, research suggests that approximately one third of patients initially exhibiting mild to moderate symptoms continue to report troubling symptoms, with 76% of survivors experiencing at least one symptom, most commonly fatigue, muscle soreness and mental health issues, 6 months after exposure.^[^ ^19^ ^]^ The reason behind the lasting effects is largely unknown, but thought to be resultant of an effect observed in other viruses, known as ‘post viral syndrome’.^[^ ^20^ ^]^ Although to date the most devastating features of COVID‐19 infection have manifested in the lungs, all organs are potentially affected by the virus. Table 2 adapted from a recent review from Wang et al., which focuses exclusively on research surrounding the long‐term effects of the COVID‐19 virus, outlines the various potential side effects on organs and tissues long after the virus has gone.^[^ ^20^ ^]^

**TABLE 2:** Potential long‐term effects of COVID‐19 exposure

Organs effected	Physical manifestation
Lungs	Pneumonia, pulmonary fibrosis, dyspnea
Brain	Meningitis, encephalitis, myelitis, acute disseminated encephalomyelitis, stroke
Endocrine	Pancreatic, thyroid, adrenal and pituitary disfunction and abnormal hormone secretions
Kidney	Cellular damage, mild proteinuria, acute kidney injury
Reproductive	Premature birth, fetal distress, and premature rupture of fetal membranes, oligospermia, orchitis, erectile dysfunction
Heart	Myocardial injury, myocarditis, acute coronary syndrome, acute myocardial infarction, cardia arrythmia, heart failure
Intestines	Appetite loss, vomiting, diarrhea, disturbance to gut flora, associated liver dysfunction and opportunistic infection
Muscles	Hypoxia and ischemia leading to myalgia and painful joints

COVID‐19 has mutated a number of times and according to the Center's for Disease Control and Prevention, includes alpha, beta, delta, gamma, epsilon, eta, iota, kappa, mu, omicron, and zeta variants. However, it is the recently identified (B.1.1.529) omicron variant in South Africa and delta variants. that are currently causing the greatest concern.^[^ ^21^ ^]^ Omicron was first identified in November 2021, although given the close resemblance to the alpha variant, it is postulated that the omicron variant has been circulating for longer, and gone largely unmanaged due to factors such as insufficient testing and poor immunization quotas.^[^ ^22^ ^]^ South Africa, the first omicron hub had previously reported high rates of transmission for the beta variant, with ∼50% increases in daily infection rates over a 100‐day period. This number rose considerably with the delta strain of the virus (∼80%). However, within as little as 25‐days the omicron's total daily infection rates soared to approximately 90%.^[^ ^2^ ^]^ Notably, researchers have identified 32 mutations to the spike protein in the omicron variant, sixteen more than the delta variant.^[^ ^22^, ^23^ ^]^ However, how this will impact transmission and pathogenicity remains to be fully determined. Initially, the rest of the world was being hit with the effects of the delta strain which was presenting with peak viral loads in vaccinated populations proportional to unvaccinated people.^[^ ^21^ ^]^ Moreover, the delta variant was spreading through, and infecting whole households of fully vaccinated occupants.^[^ ^21^ ^]^ However, when the omicron virus did subsequently arrive in these other countries, the data showed that people infected with the omicron variant had a 50% greater chance of infecting household members than those diagnosed with the delta variant and that 3‐dose vaccine exposure did not reduce this number.^[^ ^24^ ^]^ Since the initial discovery, ongoing analysis of omicron has identified two sublineages composed of BA.1, BA.1 with an R346K mutation (BA.1+R346K, also known as BA.1.1) and B.1.1.529.2 (BA.2).^[^ ^25^ ^]^

### 1.2 COVID‐19 virions and their ability to gain cellular entry

**FIGURE 1:**
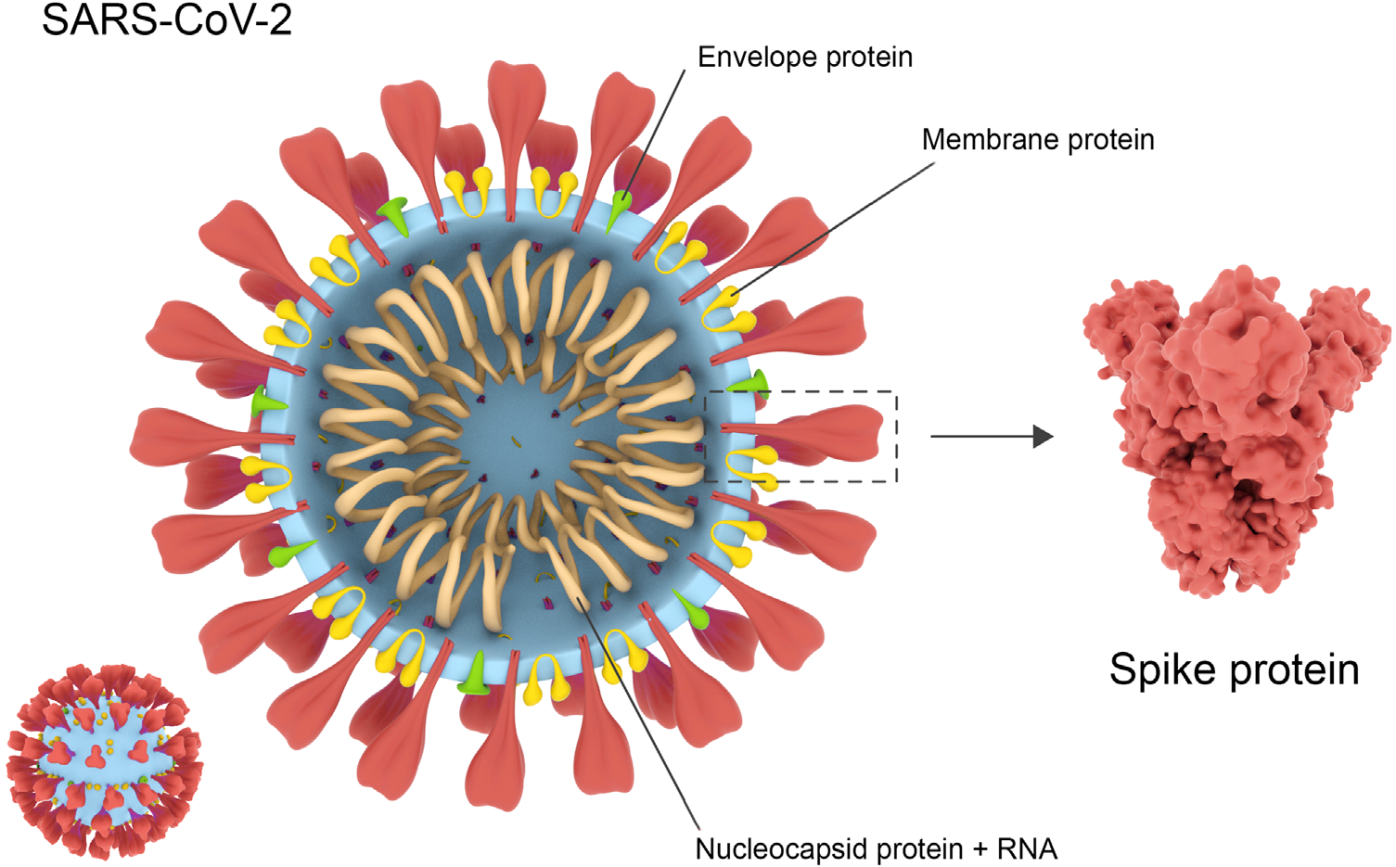
COVID‐19 structural identity. The spike protein (red) jutting out from the main body of the virion is used to attach to recipient cells. The envelope and membrane (green and yellow) provide structure to the virion, whilst the nucleocapsid protein provides a protective casing around the viral RNA (Blue and brown, respectively). Reproduced with permission.^[^ ^112^ ^]^ Copyright 2021, Rahbar Saadat

Each COVID‐19 virus particle measures approximately 130 nm in length and is developed from four structural proteins and sixteen non‐structural proteins.^[^ ^4a^ ^]^ Three of the structural proteins form a flexible lipid membrane envelope containing notably spiked protrusions jutting outwards towards the biological environment. The fourth protein builds a protective nucleocapsid that encases the COVD‐19 genome and helps transport it into a host cell (Figure 1).^[^ ^4^, ^26^ ^]^ Non‐structural proteins are deployed during RNA processing, signalling, host cell modification and replication.^[^ ^4a^ ^]^ The whole of the COVID‐19 genome comprises of just a single molecule of capped and polyadenylated ribonucleic acid (RNA). The single strand of RNA is approximately 26–32 kb long and consists of six major open‐reading frames that are common to the Betacoronavirus group.^[^ ^1^, ^26^, ^27^ ^]^ Cellular entry for Coronaviridae viruses including COVID‐19 is dictated by the glycoprotein spikes on the surface of the virions.^[^ ^28^ ^]^ The S gene responsible for the production of the spike is the only gene that is markedly different to that of other COVID's in the family.^[^ ^1^ ^]^ For successful entry into a host cell, the COVID‐19 spikes must transition from a pre‐fusion to post‐fusion conformation.^[^ ^29^ ^]^

**FIGURE 2:**
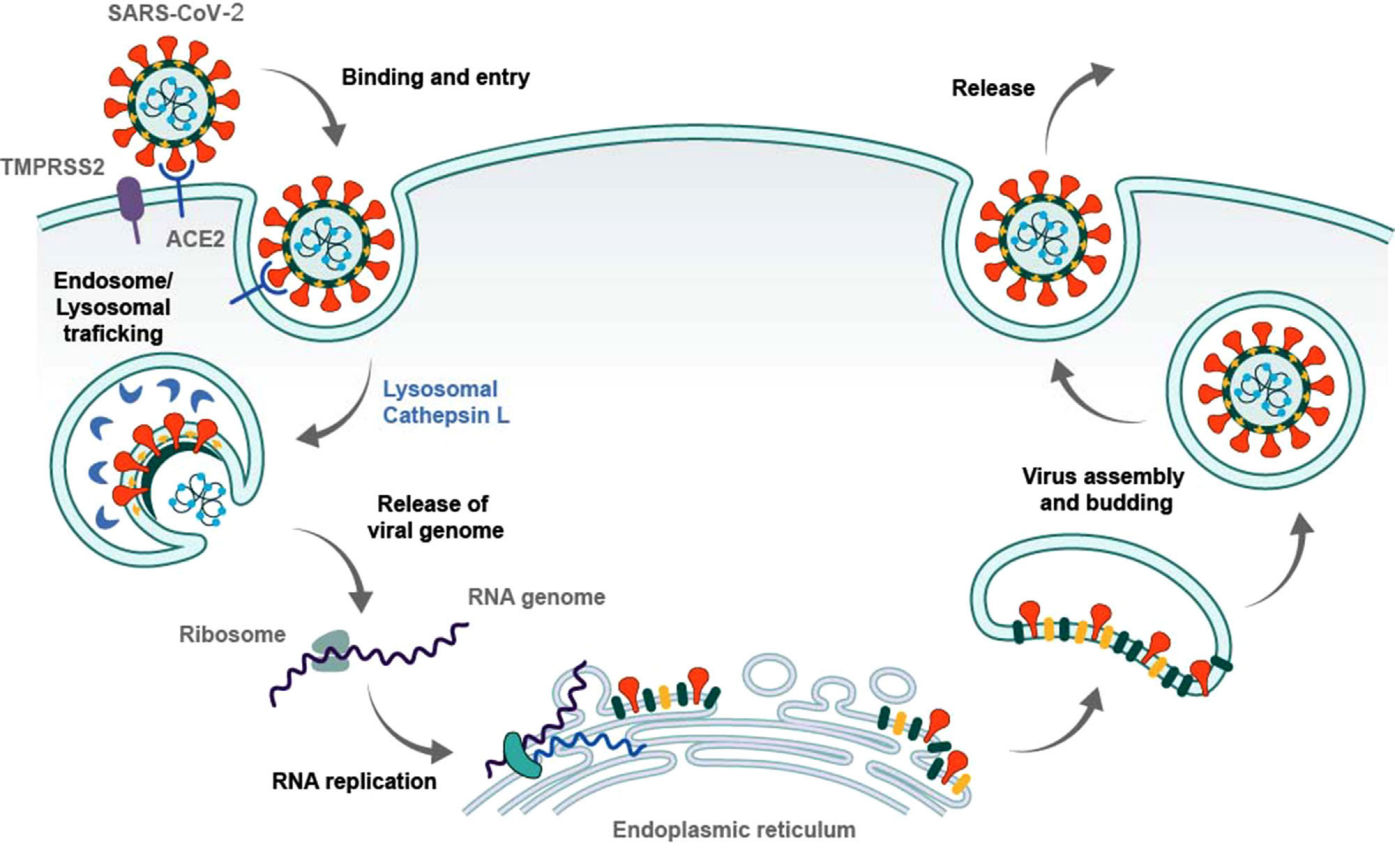
Schematic describing cycle of COVID‐19 replication. Fusion of the COVID‐19 virion to a cell membrane is primarily through the spike proteins and ACE2 receptors, with assistance from membrane protein TMPRSS2. Binding and subsequent endosomal uptake ensures the viral RNA is released from the viral envelope and nucleocapsid into the cytoplasm. In the cytoplasm, the viral RNA is translated by the intracellular ribosomes. This results in production of sixteen non‐structural proteins and subsequent hijacking of endoplasmic reticulum (ER)‐derived membranes. Fully developed structural proteins assemble in the ER‐Golgi intermediate compartment to form the nucleocapsid and envelope, encasing the genomic RNA prior to cellular release through exocytosis. Reproduced with permission.^[^ ^113^ ^]^ Copyright 2020, Cell Press

Access into the cell, is primarily facilitated by angiotensin‐converting enzyme 2 (ACE2) present on the cell surface, the spikes binding with high affinity to the ACE2 receptors.^[^ ^26^ ^]^ Cleavage of the spike protein required prior to virion entry, is performed by the peptidase function of ACE2. Cathepsin L, and serine protease TMPRSS2, are also utilised during ACE2 mediated internalisation of the majority of COVID‐19 variants.^[^ ^26^, ^30^ ^]^ ACE2 expression is highest on hepatocytes, blood vessel endothelial cells, gastrointestinal epithelial cells, epithelial cells that line the nasal cavity and type II alveolar cells in lungs and as such, all of these cells are particularly at risk from COVID‐19 attack.^[^ ^31^ ^]^ In particular, single‐cell RNA‐sequencing data analysis suggests that the high expression of ACE2 found on type II alveolar cells explains why severe pneumonia is a rapidly evolving pathology in patients with COVID‐19 infection.^[^ ^31^ ^]^ However, unlike all other COVID‐19 variants, the omicron variant is thought to endocytose into cells in the absence of TMPRSS2 and this is suggested to be why the omicron infection presents differently and is not highly specific to the lungs.^[^ ^32^ ^]^ Moreover, in this variant, the virus has an increased affinity with ACE2, which may explain the increase in transmissibility.^[^ ^33^ ^]^ Once inside of a host cell, the cycle of replication is achieved through the hijacking of normal cellular functions that culminate in the production of viral proteins and the formation of virions that bud from the cell membrane, ready to infect the next cell (Figure 2).

## 2 CURRENT COVID‐19 THERAPEUTIC STRATEGIES

### 2.1 Vaccines

The global escalation of COVID‐19 has resulted in a flood of potential vaccine candidates going at an unprecedented speed through clinical trials. To date, there are seventeen vaccines that the World Health Organization (WHO) has under evaluation (Table 3). The most widely utilised vaccines for COVID‐19 so far have been Pfizer BNT162b2 and Oxford University‐AstraZeneca AZD1222, respectively.^[^ ^34^ ^]^ Pfizer BNT162b2 is a ribonucleic acid (RNA) based vaccine, made, and synthesised in the absence of viral particulate. Importantly, it has been suggested that because supply chains for upstream and downstream processes are identical in RNA vaccines, manufacturing is quicker and notably easier than for protein vaccines.^[^ ^29^ ^]^ BNT162b2 entry into cells is reliant on positively charged lipid nanoparticles to deliver the messenger RNA (mRNA) across the negatively charged cell membrane and into the cytoplasm. Once inside the cytoplasm, the mRNA is translated by intracellular ribosomes, into a functional protein.^[^ ^35^ ^]^ The protein encoded in the mRNA of the BNT162b2 vaccine is for the COVID‐19 spike protein. The Oxford University‐AstraZeneca's vaccine is also built around targeting the COVID‐19 spike protein. It is a chimpanzee adenovirus vectored vaccine (ChAdOx1 nCoV‐19) referred to as AZD1222. AZD1222 is a replication‐deficient viral vector that expresses the full‐length DNA for the COVID‐19 spike protein. The viral vector works as a delivery vehicle, transporting the DNA into a cell's cytoplasm. Once inside, the DNA is delivered to the cell nucleus where it is subsequently catalysed into mRNA that codes for the COVID‐19 spike protein by RNA polymerase II.^[^ ^36^ ^]^ Vaccines that produce the COVID‐19 spike protein in the absence of COVID‐19 infection are seen by the immune system as foreign evaders and so the immune system begins production of COVID‐19 spike‐specific antibodies. Subsequently, if the COVID‐19 spike protein antibodies are presented with COVID‐19 virions at a later date, the antibodies will initiate a pre‐programmed immune response that prevents the viral spike from engaging with cell receptor ACE2.^[^ ^37^ ^]^ The inability of spikes to bind to ACE2 receptors subsequently prevents entry into host cells that are needed for the virus to replicate.

**TABLE 3:** World Health Organization list of vaccines under evaluation

Manufacturer	Vaccine name	Platform	Approval body
BioNTech Manufacturing GmbH	BNT162b2/COMIRNATY Tozinameran (INN)	Nucleoside modified mNRA	EMA and USFDA
AstraZeneca, AB	AZD1222 Vaxzevria	Recombinant ChAdOx1 adenoviral vector	EMA, MFDS KOREA, Japan MHLW/PMDA, Australia TGA
Serum Institute of India Pvt. Ltd	Covishield (ChAdOx1_nCoV‐19)	Recombinant ChAdOx1 adenoviral vector	DCGI
Janssen–Cilag International NV	Ad26.COV2.S	Recombinant, replication incompetent adenovirus type 26 (Ad26)	EMA
Moderna Biotech	mRNA‐1273	mNRA‐based vaccine encapsulated in lipid nanoparticle (LNP)	USFDA
Beijing Institute of Biological Products Co., Ltd. (BIBP)	SARS‐CoV‐2 Vaccine (Vero Cell), Inactivated (lnCoV)	Inactivated, produced in Vero cells	NMPA
Sinovac Life Sciences Co., Ltd	COVID‐19 Vaccine (Vero Cell), Inactivated/ CoronavacTM	Inactivated, produced in Vero cells	NMPA
Gamaleya National Centre	Sputnik V	Human Adenovirus Vector‐based	Russian NRA
Bharat Biotech, India	SARS‐CoV‐2 Vaccine, Inactivated (Vero Cell)/ COVAXIN	Whole‐Virion Inactivated Vero Cel	DCGI
Sinopharm / WIBP	Inactivated SARS‐CoV‐2 Vaccine (Vero Cell)	Inactivated, produced in Vero cells	NMPA
CanSinoBio	Ad5‐nCoV	Recombinant Novel Coronavirus Vaccine (Adenovirus Type 5 Vector)	NMPA
Novavax	NVX‐CoV2373/Covovax	Recombinant nanoparticle prefusion spike protein formulated with Matrix‐M™ adjuvant.	EMA
Sanofi	CoV2 preS dTM‐AS03 vaccine	Recombinant, adjuvanted	EMA
Serum institute of India PYT LTD	NVX‐CoV2373/Covovax	Recombinant nanoparticle prefusion spike protein formulated with Matrix‐M™ adjuvant.	DCGI
Clover Biopharmaceuticals	SCB‐2019	Novel recombinant SARS‐CoV‐2 Spike (S)‐Trimer fusion protein	NMPA
Urevac	Zorecimeran (INN)	mNRA‐based vaccine encapsulated in lipid nanoparticle (LNP)	EMA
Zhifei Longcom, China	Recombinant Novel Coronavirus Vaccine CHO Cell)	Recombinant protein subunit	NMPA

The roll out of vaccines has without doubt saved many lives. For instance, it has been documented that the COVID‐19 vaccination programmes have prevented an estimated 469,186 deaths in thirty‐three countries for older populations considered to be most likely to succumb to COVID‐19 infection.^[^ ^38^ ^]^ However, COVID‐19 vaccines have also been linked to some very severe side effects. Most notably, AZD1222, marketed as safe and affordable to produce, easy to store (2–8°C) and with a shelf life of approximately 6 months, was the first to come under scrutiny. Firstly, this vaccine was only available to people over the age of 50, as thromboembolic events in younger women had been reported in clinical trials.^[^ ^39^ ^]^ Then, not long after its roll out, the safety of AZD1222 was again being called into question in the media after there were reports of deaths occurring in patients who had received their first initial dose of the vaccine. The reputation of the AZD1222 vaccine was severely tarnished and countries began temporarily suspending its use.^[^ ^40^ ^]^ These findings were further compounded by reports of neurological complications including Guillain‐Barre syndrome, and myasthenic disorders.^[^ ^41^ ^]^ Pfizer's BNT162b2 has also come under scrutiny. Firstly, needing to be maintained at −80°C during transportation makes delivery and distribution challenging.^[^ ^42^ ^]^ Moreover, reports suggested that a dose of the vaccine potentially increased the risk of Bell's palsy and haemorrhagic stroke.^[^ ^41^ ^]^ An additional study of BNT162b2 from a data set involving more than 2.4 million vaccinated persons identified that vaccination with BNT162b2 was strongly associated with elevated risk of myocarditis, lymphadenopathy, appendicitis.^[^ ^43^ ^]^ Importantly, if patients had contracted COVID‐19 and were infected at the time of vaccination, this substantially increased the risk of developing myocarditis and other morbidities, including pericarditis, arrhythmia, deep‐vein thrombosis, pulmonary embolism, myocardial infarction, intracranial haemorrhage, and thrombocytopenia.^[^ ^43^ ^]^

On average, vaccine development takes decades before a product reaches the market. However, in the case of COVID‐19, it has in some instances taken little more than 3 months to reach Phase I trials, with Phase III trials subsequently taking place just a few months later.^[^ ^44^ ^]^ It is clear that when it comes to COVID‐19 vaccines, we have been able to fast track through some of the bureaucracy that has previously slowed down drug developmental pathways and this has saved many lives. However, some may question as to whether the harsh side effects identified, and deaths that have occurred after the rollout are an acceptable outcome in the bigger picture, even though the numbers have clearly shown that benefits have outweighed the risks. Moving forwards, it remains imperative that in this sped up process, we do not end up repeating past mistakes during development, such as those of the infamous ‘Cutter Incident’ and vaccine initiation of antibody‐dependent enhancement, identified in a potential vaccine made to treat HIV patients, or lastly, the onset of vaccine‐associated enhanced respiratory disease demonstrated in a previously developed measles vaccine.^[^ ^29^, ^45^ ^]^ As countries go ‘all in’ with a third dose of COVID‐19 vaccines in an attempt to curb the effects of the delta and omicron variants, it is worth remembering that if patients had contracted COVID‐19 and were infected at the time of vaccination, this substantially increased the risk of developing harsh side effects.^[^ ^43^ ^]^ Furthermore, recent studies have identified a loss of neutralizing activity with current vaccines in the newly identified omicron sublineages.^[^ ^25^ ^]^ What omicron has taught us is that we do not really know how long it will be before sensitivity to the current vaccines stop altogether. We also do not know the full extent to which the COVID‐19 virus impacts health long‐term, or how repeated exposure to COVID‐19 vaccines may impact overall health long‐term, and obviously this aspect we will not know until years to come. Therefore, it remains vital that additional single dose vaccines, as suggested by researchers including Pilkington et al., as well as alternative approaches that help stop the spreading of the COVID‐19 virus and prevent COVID‐19 symptoms from becoming life‐threatening, are produced and commercially available to all.^[^ ^46^ ^]^ In this respect, antiviral approaches present as a good way to modulate COVID‐19 virulence and help negate the notable side effects occurring with COVID‐19 vaccines.

### 2.2 Antivirals

Since the beginning of the COVID‐19 pandemic, there has been huge interest in the design of COVID‐19 antivirals that either stop viral entry into a host cell or hinder replication following entry. Clinically approved antivirals to date fit within two therapeutic classes, antibody‐based and drug based.

#### 2.2.1 Antibody approved approaches to target COVID‐19

According to Asdaq et al., over 100 patent applications detailing the use of monoclonal antibodies against COVID‐19 have been registered.^[^ ^47^ ^]^ As it stands, there are two antibody‐based strategies being tested, the first strategy is where antibodies are being used to target and neutralise COVID‐19 and the second strategy aims to diminish the inflammatory effect caused by the infection.^[^ ^48^ ^]^ For production of neutralising antibodies, human B‐cell cloning has proven most successful.^[^ ^49^ ^]^ AstraZeneca's antibody treatment AZD7442 (Evusheld) was proving to be a novel front runner as a potent COVID‐19 antibody treatment. AZD7442 was recently approved by the FDA, after being tested in two separate trials where results proved it to be 83% effective at reducing symptoms and when taken 3 days after symptom onset, reduced the chances of patients becoming severely ill, and even reduced the risk of death by 88%.^[^ ^50^ ^]^ However, these studies were performed before the identification of omicron. AZD7442 is a formulation of two antibodies, tixagevimab and cilgavimab, produced through human B‐cell cloning from patients exposed to the COVID‐19 virus.^[^ ^51^ ^]^ A list of potential neutralising antibody candidates is shown in Table 4. As demonstrated, all candidate antibodies work by targeting the spike protein.

**TABLE 4:** List of neutralising antibodies in use against COVID‐19

Name	Antibody class and target	Effective against BA.2 sublineage of omicron	Combination recommendation	Type of approval	Registered clinical trials
Casirivimab	Anti‐spike monoclonal	No	Imdevimab	Emergency use authorisation	NCT05092581; NCT04992273; NCT05149300; NCT04840459; NCT05074433; NCT04425629; NCT04790786; NCT04852978; NCT05081388
Imdevimab	Anti‐spike monoclonal	No	Casirivimab	Emergency use authorisation	
Bamlanivimab	Anti‐spike monoclonal	No	Etesevimab	Emergency use authorisation	NCT04840459; NCT04796402 NCT04840459; NCT04748588; NCT04790786; NCT04518410; NCT04885452
Etesevimab	Anti‐spike monoclonal	No	Bamlanivimab	Emergency use authorisation	
Tixagevimab	Anti‐spike monoclonal	No	Cilgavimab	Emergency use authorisation	NCT04625972; NCT04625725; NCT04723394; NCT04518410; NCT04501978; NCT04315948
Cilgavimab	Anti‐spike monoclonal	Yes	Tixagevimab	Emergency use authorisation	
Sotrovimab	Anti‐spike monoclonal	No		Emergency use authorisation	NCT05124210; NCT05144178; NCT04913675; NCT04779879; NCT05135650; NCT04748588; NCT04790786
Bebtelovimab	Anti‐spike monoclonal	Yes		Emergency use authorisation	NCT04634409

It is also worth noting that for severely affected COVID‐19 patients alone, an estimated 2.5 million doses of antibody therapy would be needed globally every month.^[^ ^49^ ^]^ This is a colossal amount that even if production was continuous in multiple sites, these dosing numbers could not be met.^[^ ^49^ ^]^ Also, the production of antibody‐based approaches for all diseases is notoriously expensive and may not be affordable to low‐income countries.^[^ ^52^ ^]^ For instance, the company behind production of the cocktail administration of casirivimab and imdevimab, Regeneron in their recent press release quoted the US government $2,100 per dose of this COVID‐19 antibody treatment.^[^ ^52^ ^]^ It would also now seem that this expense is meaningless, as casirivimab and imdevimab are not effective against omicron.^[^ ^53^ ^]^ However, at least in terms of cost, there is hope that antibody production can be made cheaper in years to come. This hope comes from the development of small antibody‐like structures called nanobodies. The idea of nanobodies is based on naturally occurring antibodies found in llamas and alpacas.^[^ ^54^ ^]^ Nanobodies produced in bacterial cells are less expensive to grow and maintain than regular human antibodies and importantly, FDA approval has already been given to one type of nanobody (caplacizumab), designed to treat rare blood clotting disorder, thrombotic thrombocytopenic purpura.^[^ ^55^ ^]^ Researchers have already applied this novel technology to COVID‐19, producing a nanobody therapy they have named Tyson that also targets the spike protein.^[^ ^54^ ^]^ However, Tyson is in early development and is yet to reach in vivo investigations. Using nanobody technology, researchers are also hoping to design an intranasal version that can easily be administered directly into the nasal cavity and lungs.^[^ ^54^ ^]^ But again, this is a long way off from reaching the clinic.

#### 2.2.2 Drug approved approaches to target COVID‐19

The majority of drugs approved for COVID‐19 are those which have been repurposed. Drug repurposing refers to the identification of already approved drugs and investigating them in a disease outside of their original intended use.^[^ ^56^ ^]^ The main advantage to drug repurposing is that the drug is less likely to fail due to problems with safety, as the toxicity profile and any side effects have already been established in previous studies.^[^ ^57^ ^]^ Drugs repurposed in an attempt to curb COVID‐19 spread and the severity of symptoms include those that inhibit the viral replication of COVID‐19 through prevention of Gag/Pol polyprotein cleavage or hindering RNA‐dependent RNA polymerase action, and those that prevent viral entry.^[^ ^58^ ^]^ Drugs used to inhibit viral replication include, Ritonavir, lopinavir and darunavir; antivirals initially designed to treat HIV/AIDS.^[^ ^59^ ^]^ Whilst others, including favipiravir, were produced in Japan as an alternative to resistant influenza infection and ribavirin was developed to treat respiratory syncytial virus (RSV) infection, hepatitis C and some viral haemorrhagic fevers.^[^ ^60^ ^]^ Remdesivir, also fitting within this inhibitory group is the only drug in its class that was produced exclusively as a COVID‐19 antiviral. Remdesivir was a promising early drug candidate that was rushed through trials and into clinical use.^[^ ^59a^ ^]^ Unfortunately, it was later proven to be ineffective at preventing deaths from occurring.^[^ ^61^ ^]^ Even so, because it was demonstrated to speed up the recovery process, Remdesivir is still listed for use by the National Institute of Health, alongside antiparasitics, Ivermectin and Nitazoxanide and, is currently in clinical trials for pregnant COVID‐19 positive participants.^[^ ^62^ ^]^ Repurposed drugs that have been clinically assigned to prevent viral entry include anti‐malarial drugs, Chloroquine and hydroxychloroquine, and influenza antiviral, arbidol.^[^ ^63^ ^]^ Lastly, one of the more unusual choices of drugs that have shown promising results in a recent Brazilian study is antidepressant formulation, fluvoxamine. Although the mechanism by which fluvoxamine works against COVID‐19 is not fully understood, the findings of the study demonstrated that when fluvoxamine is administered to high‐risk outpatients with early diagnosed COVID‐19, the drug reduced the need for hospitalisation.^[^ ^64^ ^]^

Novel antiviral pill formulae are the latest addition to the COVID‐19 drug arsenal and have been developed by Merck and Pfizer, respectively. The pills present as an easy way for patients to be treated early on in the infection process and do not require a hospital visit. This is important, as it has been suggested that early intervention may prevent the development of inflammation, associated with severe illness.^[^ ^65^ ^]^ On 4 November 2021, Molnupiravir, developed in partnership with Merck and biotech company Ridgeback Biotherapeutics, was approved in the United Kingdom for the treatment of COVID‐19 patients with mild to moderate symptoms, with the US following suit shortly after.^[^ ^66^ ^]^ Authorisation for use came following clinical trials which demonstrated that Molnupiravir intervention halved the risk of patients needing to be hospitalised.^[^ ^67^ ^]^ Molnupiravir is an orally administered ribonucleoside antiviral pro‐drug that causes mutations to be added into the viral genome during the process of replication. Upon metabolisation, Molnupiravir converts into β‐d‐N^4^‐hydroxycytidine (NHC).^[^ ^68^ ^]^ NHC is subsequently slotted into the viral genome by the viral enzyme, RNA‐dependant RNA polymerase. The introduction of NHC causes an abundance of errors that overwhelms the genome, preventing the virus from replicating further.^[^ ^69^ ^]^ Paxlovid by Pfizer has also received notoriety after clinical trials identified that when COVID‐19 patients received Paxlovid within 3 days of contracting the virus, the number of COVID‐19 patients needing hospital admission was reduced by approximately 90%, compared to the placebo group.^[^ ^70^ ^]^ Paxlovid, also administered orally, is a formula that consists of a compound called PF‐07321332 and ritonavir, an antiretroviral used to treat HIV Paxlovid works by disrupting the COVID‐19 replication process through binding to the 3CL‐like protease enzyme, required for normal function and replication of the COVID‐19 virions.^[^ ^70^ ^]^ Clinical trial data for Molnupiravir or Paxlovid respectively demonstrated that both antivirals were well received by cohort participants, with no significant side effects being reported.^[^ ^67^ ^]^ However, due to the individual targeting mechanisms of both drugs, there remain some concerns regarding their safety. For instance, due to the therapeutic effect of Molnupiravir, which is to create mutations within the viral genome, one study suggests that this mechanism can be translated into human cells and their DNA, subsequently causing mutations to erupt within the human genome.^[^ ^68^ ^]^ This is particularly troubling given that genomic instability is a well‐established hallmark of cancer pathogenesis.^[^ ^68^, ^71^ ^]^ Even more troubling are the recent findings that the omicron variant has mutated its RdRp meaning that Molnupiravir may not be as viable in treating the omicron variant. Although research presented in The New England Journal of Medicine suggests that this is not the case.^[^ ^53^, ^72^ ^]^ Paxlovid on the other hand requires processing by the liver and therefore may not be suitable for those with liver ailments. Furthermore, it could potentially affect other medications metabolised by the liver.^[^ ^73^ ^]^ Therefore, extra caution has been proposed when administering to patients with cardiac conditions and patients receiving either pain or immunosuppressive drugs. Even though no deaths were recorded in either of the separate clinical trials, disappointingly, neither Molnupiravir nor Paxlovid clinical trial cohorts were sufficient in number to identify whether they could in fact prevent deaths altogether. Lastly, manufacturers have asked for caution to be taken when administering Molnupiravir during pregnancy as there is insufficient data to prove that treatment is not damaging to the fetus.^[^ ^74^ ^]^

#### 2.2.3 The use of decoys as neutralizing agents to treat COVID‐19

**FIGURE 3:**
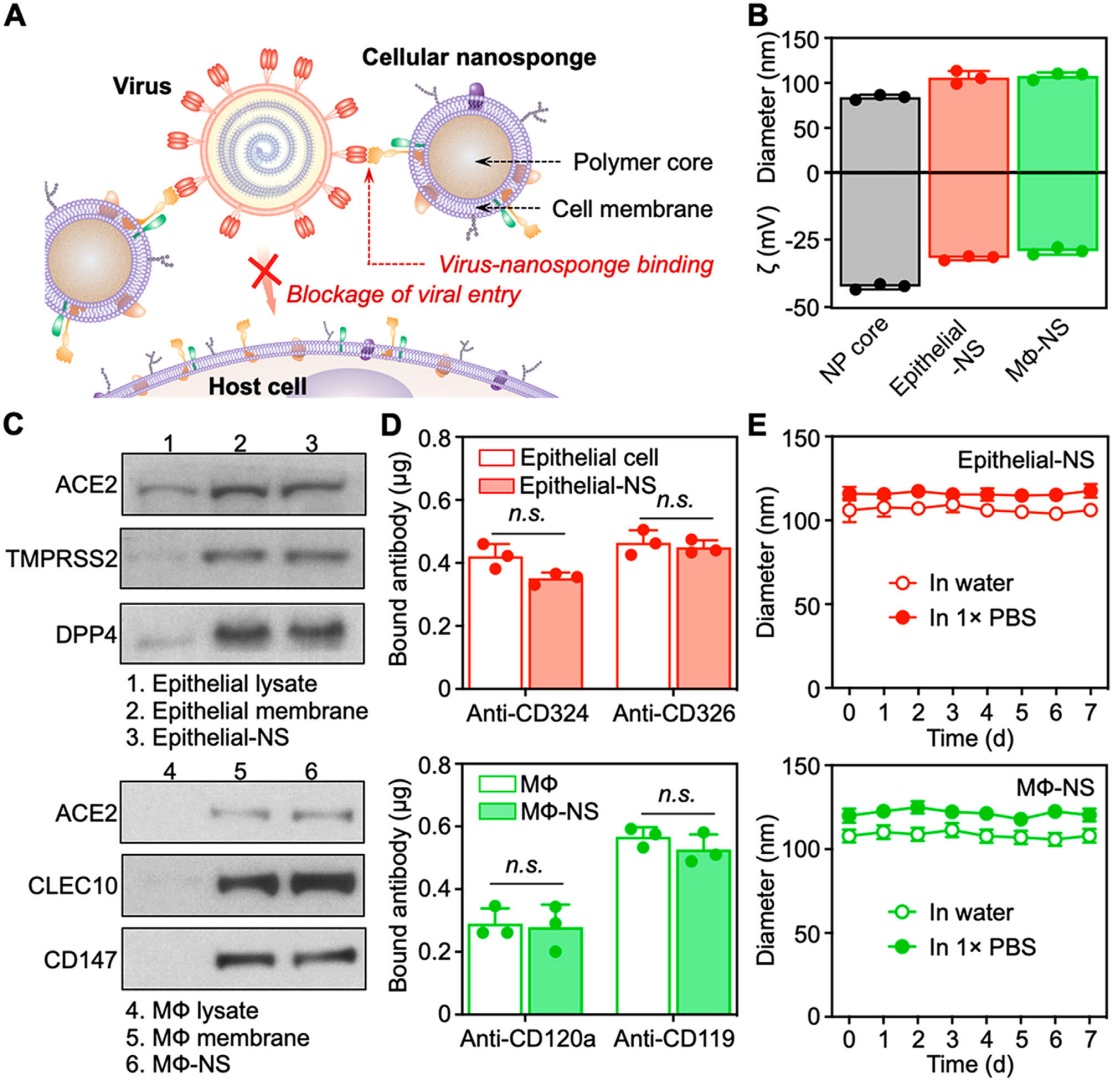
Nanosponges act as decoys that bind with COVID‐19 and block viral entry into recipient cells, in vitro characterisation. (A) Schematic demonstrating nanosponge mechanism of action. (B) Dynamic light scattering measurements of hydrodynamic size (diameter, nm) and surface zeta‐potential (ζ, mV) of polymeric NP cores before and after coating with cell membranes. (C) Western blotting analysis of cell lysate, cell membrane vesicles, and cellular nanosponges. (D) Antibody binding assay results. (E) Stability measured over 7‐days. Reproduced with permission.^[^ ^75^ ^]^ Copyright 2020, American Chemical Society

An interesting alternative COVID‐19 antiviral approach is to use decoys as neutralizing agents, where focus is placed on the host cell instead of the virus itself. For instance, Zhang et al., have developed nanosponges made by encasing a polymer nanoparticle core with a membrane coat. The membranes are sourced from human lung epithelial cells that are highly susceptible to COVID‐19 infection and macrophage that have previously been significantly associated with hyper‐inflammation and the overall pathology of COVID‐19 and other COVID viruses.^[^ ^75^ ^]^ The results of this study show that nanoponges act as decoys that bind with COVID‐19 and block viral entry into recipient cells. Following an in‐depth in vitro characterisation (Figure 3), the results from the in vivo study demonstrated that nanosponges neutralise the viral activity and subsequently viral load both in early and late‐stage CPVID‐19 infection. Furthermore, they can effectively neutralise inflammation associated with COVID‐19.^[^ ^75^ ^]^ Li et al., have also used a decoy method in their recent study. In this example, nanodecoys were derived from human lung spheroid cells and delivered intranasally, with the results demonstrating that the nanodecoys remained within the lungs for approximately 72 h. This resulted in a more rapid clearance of the COVID‐19 virus from the lungs without any visible toxicity. Four doses of the nanodecoy were shown to both clear the lungs of infection and restrict lung injury in a non‐human primate model of COVID‐19.^[^ ^76^ ^]^ Lastly, Rao et al., have proposed the use of decoy nanoparticles aimed at adsorbing to COVID‐19 virions and pro‐inflammatory associated cytokines. In this study, the authors demonstrate that the decoy nanoparticles labelled with an abundance of the ACE2, and cytokine receptors provide an alternative for the COVID‐19 virus to bind to, which significantly inhibits viral replication. Moreover, biding of cytokines including IL‐6 and GM‐CSF, resulted in suppression of inflammation, and prevented subsequent immune activated lung damage in an acute lung injury mouse model.^[^ ^77^ ^]^

In summary, the discovery and implementation of novel antivirals should above all be safe, display minimal side effects and be therapeutics that are easily administered all to patients regardless of any comorbidities. Although pre‐clinical approaches including the use of decoys look promising, they are still a way off from reaching patients in the clinic and many drugs, including Molnupiravir and Paxlovid are not suitable for all patients. We, therefore, conclude that alternative strategies are still very much needed. RNA interference antivirals designed to prevent the synthesis of essential viral proteins, have the potential to be minimally toxic and could present as a novel strategy for the ongoing control of severe COVID‐19 infection, even in pregnant patients and those with underlying health issues.

## 3 COVID‐19 siRNA antiviral approaches

Antiviral therapeutics that display few side effects and can directly target the ever‐evolving pathogenicity of the COVID‐19 virus are urgently needed. siRNA is a rapidly advancing technology that can be used to easily keep up with the mutational force of COVID‐19 variants because siRNA sequences can potentially be quickly designed for any alterations identified in the encoded target COVID‐19 genes.^[^ ^78^ ^]^ In terms of manufacturing, the synthesis of siRNA is a fast and relatively straightforward process that can be successfully upscaled.^[^ ^79^ ^]^ Encouragingly, siRNA can be carefully designed to limit off‐target toxicity and has proven in general to be safe. Additionally, siRNA presents a robust way to target host receptors most notably ACE2 as demonstrated by Friedrich et al., thus preventing viral entry, and investigating and exploring newly emerging COVID‐19 gene targets.^[^ ^80^ ^]^

**FIGURE 4:**
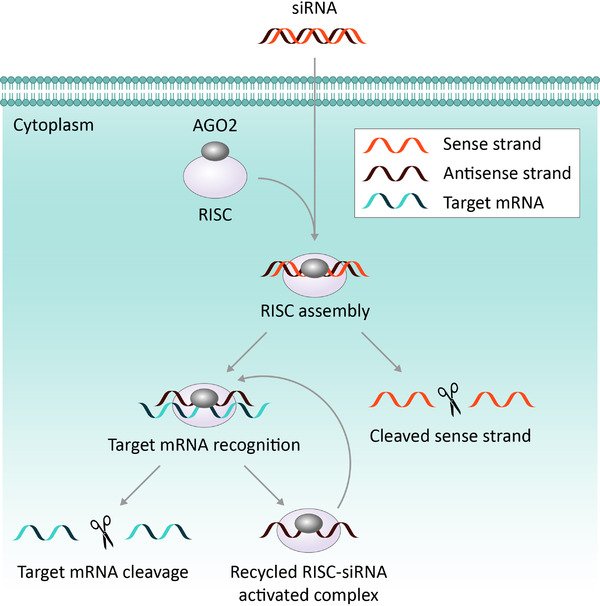
Schematic describing siRNA mediated gene silencing. The delivery of double stranded siRNA into a cell is facilitated by a nanoparticle delivery vehicle. Once inside of the cell, the siRNA binds to Argonaute 2 (ARGO2) and enters the RNA‐induced silencing complex (RISC). Inside the complex, ARGO2 cleaves the sense strand of the siRNA duplex, leaving an antisense guide strand in the now activated RISC–siRNA complex. Complementary binding between the antisense siRNA strand and the target mRNA causes breakage to occur in the reading frame for the encoded protein and subsequent degradation of the mRNA strand. Once activated, the RISC–siRNA complex can be used again to destroy identically sequenced mRNA.^[^ ^84a^ ^]^) Reproduced with permission.^[^ ^114^ ^]^ Copyright 2010, Springer Nature

RNA interference (RNAi) is a conserved mechanism initiated to defend a cell in response to number of stressors including viral attack.^[^ ^81^ ^]^ The cell does this by disposing of the harmful messenger RNA (mRNA), which prevents translation of the destructive protein encoded within it. This is contrast to mRNA vaccines that are used to encourage protein synthesis of a viral gene in order to produce an antibody‐mediated immune response.^[^ ^82^ ^]^ siRNA is a process conserved in all cells throughout the body and can be exploited to target aberrant genes that are causing a disease to propagate.^[^ ^81^ ^]^ Synthetic short interference RNA (siRNA) is a class of RNAi therapeutics that can switch off disease‐influencing genes with high specificity and low toxicity.^[^ ^81^ ^]^ Importantly, siRNA could be used to provide a kill switch to genes that produce pathogenic proteins that would otherwise be difficult to inhibit.^[^ ^83^ ^]^ The mechanisms involved in siRNA‐led gene silencing is well understood and has been described in detail in several original reviews, one of which is described here.^[^ ^84^ ^]^ A brief outline of the mechanisms involved in siRNA mediated gene silencing is illustrated in Figure 4.

The greatest challenge to therapeutic siRNA development has always been the ability to get siRNA to the diseased cells in a hostile biological environment designed to rapidly clear foreign material and then facilitate uptake by the target cells.^[^ ^85^ ^]^ Firstly, free siRNA within the body is poorly bioavailable and either quickly destroyed by RNAses present in the blood or removed promptly by the reticuloendothelial system, due to its small size (∼13 kDa).^[^ ^86^ ^]^ Second, although its small size initiates rapid removal in terms of systemic clearance, siRNA is found to be too big in size and is also negativity charged, which means that it is repelled by the negatively charged cell membrane due to electrostatic repulsion (Coulomb force), and therefore unable to enter the cytoplasm unaided.^[^ ^85^, ^87^ ^]^ siRNA, therefore, requires a positively charged (cationic) nano sized vehicle to interact with, that binds and protects it from enzyme degradation and helps drive the siRNA cargo through the cell membrane and into the cytoplasm.^[^ ^88^ ^]^ Producing a nanoparticle formula with all the prerequisite characteristics for successful biological delivery has proven challenging. The reasons behind these challenges are highly related to the positive surface charge needed to facilitate binding of the siRNA through electrostatic interaction.^[^ ^80b^ ^]^ For example, cationic nanoparticles, whether designed from organic or inorganic materials can promote biotoxicity by charge‐related disturbance affecting the membrane integrity of cells.^[^ ^80b^ ^]^ Moreover, low‐level exposure has been shown to initiate vacuolization, cause cells to shrink and interfere with routine mitosis.^[^ ^80b^ ^]^ Whereas high‐level exposure has been demonstrated to promote cell lysis and pathological cell death. At the cell membrane on entry, or once inside the cytoplasm, the positively charged ions may also interfere with proteins, which has previously been demonstrated for protein kinase C.^[^ ^89^ ^]^ Or alter the expression of genes, particularly those associated with the apoptotic pathways.^[^ ^90^ ^]^ However, a breakthrough came in 2018 with the FDA approval of the first siRNA therapeutic, produced by market leaders Alnylam. Patisiran is a nanoparticle delivered siRNA that targets Transthyretin mRNA. Patisiran is used for the treatment of polyneuropathy in people with the genetic disorder, hereditary transthyretin‐mediated amyloidosis.^[^ ^80a^ ^]^ Two more of Alnylam's siRNA therapeutics quickly followed into the clinic. Givosiran and lumasiran, used to treat acute hepatic porphyria and primary hyperoxaluria, respectively.^[^ ^91^ ^]^ As it stands, there are an additional seven siRNAs in late‐stage clinical trials for a variety of pathological conditions, as detailed in Table 5.^[^ ^92^ ^]^

**TABLE 5:** siRNA therapeutics approved or in late‐stage clinical trials

Name	Manufacturer	Disease	mRNA target	Trial status
Patisiran	Alnylam	Hereditary transthyretin mediated amyloidosis	Transthyretin (TTR)	FDA approved 2018
Givosiran	Alnylam	Acute hepatic porphyria	Aminolevulinic acid synthase 1 (ALAS1)	FDA approved 2019
Lumasiran	Alnylam	Primary hyperoxaluria type 1 (PH1)	Hydroxyacid oxidase 1 (HAO1)	FDA approved 2020
Vutrisiran	Alnylam	Hereditary transthyretin mediated amyloidosis	Transthyretin (TTR)	Phase III – NCT03759379; NCT04153149
Teprasiran	Quark‐Norvartis	Prevention of Major Adverse Kidney Events (MAKE)	p53	Phase III – NCT02610296
Inclisiran	Alnylam‐Novartis	Cardiovascular Disease	PCSK9	Phase III – NCT03397121; NCT03399370; NCT3400800
Fitusiran	Alnylam‐Sanofi Genzyme	Haemophilia A and B	Antithrombin (AT)	Phase III – NCT03417245; NCT03417102; NCT03549871; NCT03974113
Nedosiran	Dicerna‐Alnylam	Acute kidney injury	Hepatic lactate dehydrogenase (LDH)	Phase III – NCT04042402
Cosdosiran	Quark	Non‐arteritic anterior ischemic optic neuropathy (NAION)	Caspase‐2	Phase II/III – NCT 02341560
Tivanisiran	Sylentis	Dry eyes and ocular discomfort; Sjögren's Syndrome	Transient Receptor Potential Vanilloid‐1 (TRPV1)	Phase III – NCT02610296

**FIGURE 5:**
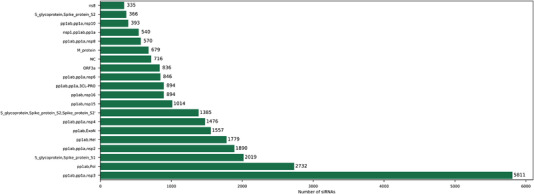
Schematic illustrating the distribution of 21 nucleotide siRNAs across the twenty‐most siRNA‐abundant COVID‐19 genes. Reproduced with permission.^[^ ^99^ ^]^ Copyright 2020, Springer Nature

Currently approved gene‐based strategies including BNT162b2 and Moderna's mRNA‐1273 vaccine have proven to be essential in the fight against COVID‐19. But these vaccines are only a small part of how gene‐based strategies can be used to better target COVID‐19. An additional promising gene‐based strategy for diagnostics and treatment is CRISPR (Clustered Regularly Interspaced Short Palin‐dromic Repeats)‐Cas (CRISPR‐associated) technology, in particular, the CRISPR‐Cas13 platform. However, CRISPR technology is beyond the scope of this current review^[^ ^93^ ^]^ siRNA‐based therapeutics may have an added advantage over these gene‐based vaccines and CRISPR technology because siRNA is not reliant on translation of mRNA. Several siRNA approaches where siRNA is delivered systemically have already been proposed for various other deadly viral diseases.^[^ ^94^ ^]^ For instance, siRNA targeting the *SUDV VP35* gene or a TKM‐Ebola siRNA cocktail of siVP35‐2 and siLpol‐2 RNAs has been delivered using lipid‐based nanoparticles for the treatment of the highly lethal Ebola virus.^[^ ^94^, ^95^ ^]^ In these current studies, Thi et al., were able to demonstrate excellent therapeutic efficacy and 100% survival in non‐human primates even after the onset of symptoms, including fever and presence of the virus in the bloodstream.^[^ ^94^, ^95^ ^]^ Additionally, Ursic‐Bedoya et al., demonstrated effective use of siRNA for the treatment of another haemorrhagic fever; Marburg virus which is also associated with severe morbidity.^[^ ^96^ ^]^ In this prospective study, gene sequences for different Marburg viral strains were analysed and siRNA was subsequently designed to target genes encoded by the 19 kb single‐stranded RNA genome. The results of the study using Guinea pigs as the experimental model, demonstrated that lipid nanoparticles delivering siRNA for target genes resulted in 60%–100% survival rate in three different viral strains of the Marburg infection.^[^ ^96^ ^]^ siRNA can also be designed and synthesised to target divergent sections of the COVID‐19 virus genome and can subsequently precision target viral mRNA sequences for degradation, thereby preventing translation of the proteins involved in replication. Also, because of target specificity and a low potential for side effects, siRNA therapeutics could be used in conjunction with approaches being applied in other therapeutic classes.^[^ ^94^, ^97^ ^]^ Despite the fast mutation rate of viruses including COVID‐19, viruses tend to contain specific genes that do not mutate at such a fast rate because the mutation could influence the virility of the virus.^[^ ^98^ ^]^ This potential pathogenic flaw can be readily exploited by siRNA‐based approaches. For instance, Medeiros et al., have recently developed a database of potential COVID‐19 targets for small interference RNA using synthetic sequences that are specific to the virus. The schematic illustration (Figure 5) details the distribution of the 21 nucleotide siRNAs spanning the twenty‐most siRNA‐abundant COVID‐19 genes.^[^ ^99^ ^]^ Several studies have detailed the potential of inhibiting COVID‐19 genes using siRNA approaches.^[^ ^58^, ^94^, ^99^, ^100^ ^]^ For instance, the RNA‐dependent RNA polymerase (RdRp) enzyme encoded by the RdRp gene was recently investigated as a promising target as an siRNA therapeutic. In this representative study, Shawan et al., used siDirect version 2.0 and RNAxs webserver to design custom siRNA against the RdRp gene and validated the sequences using the siRNAPred webserver.^[^ ^100c^ ^]^ Subsequent molecular docking and dynamic stimulation experiments proved promising and worthy of further biologically established investigations. More biologically centric investigations include that of Khaitov et al., who chose the most effective siRNA target from a panel of 15 identified in silico and tested efficacy using virally transduced HEp‐2 cells and in vivo using hamsters exposed to aerosolised COVID‐19 (strain B).^[^ ^100b^ ^]^ siRNA was delivered via cationic dendrimeric peptide KK‐46. The design of the dendrimeric peptide was founded on in silico calculations relating to the molecular properties of the complex, ensuring charge and amphiphilicity was optimal for both siRNA binding and endocytosis. siRNA delivered by the dendrimeric peptide KK‐46 was demonstrated to inhibit RNA‐dependent RNA polymerase (RdRp) and thus viral replication in vitro and significantly reduce the virus titre and lung inflammation in vivo.^[^ ^100b^ ^]^ Importantly, on the back of these impressive results, a permission to begin clinical trials for siR‐7‐EM/KK‐46 in Russia has been granted.

**FIGURE 6:**
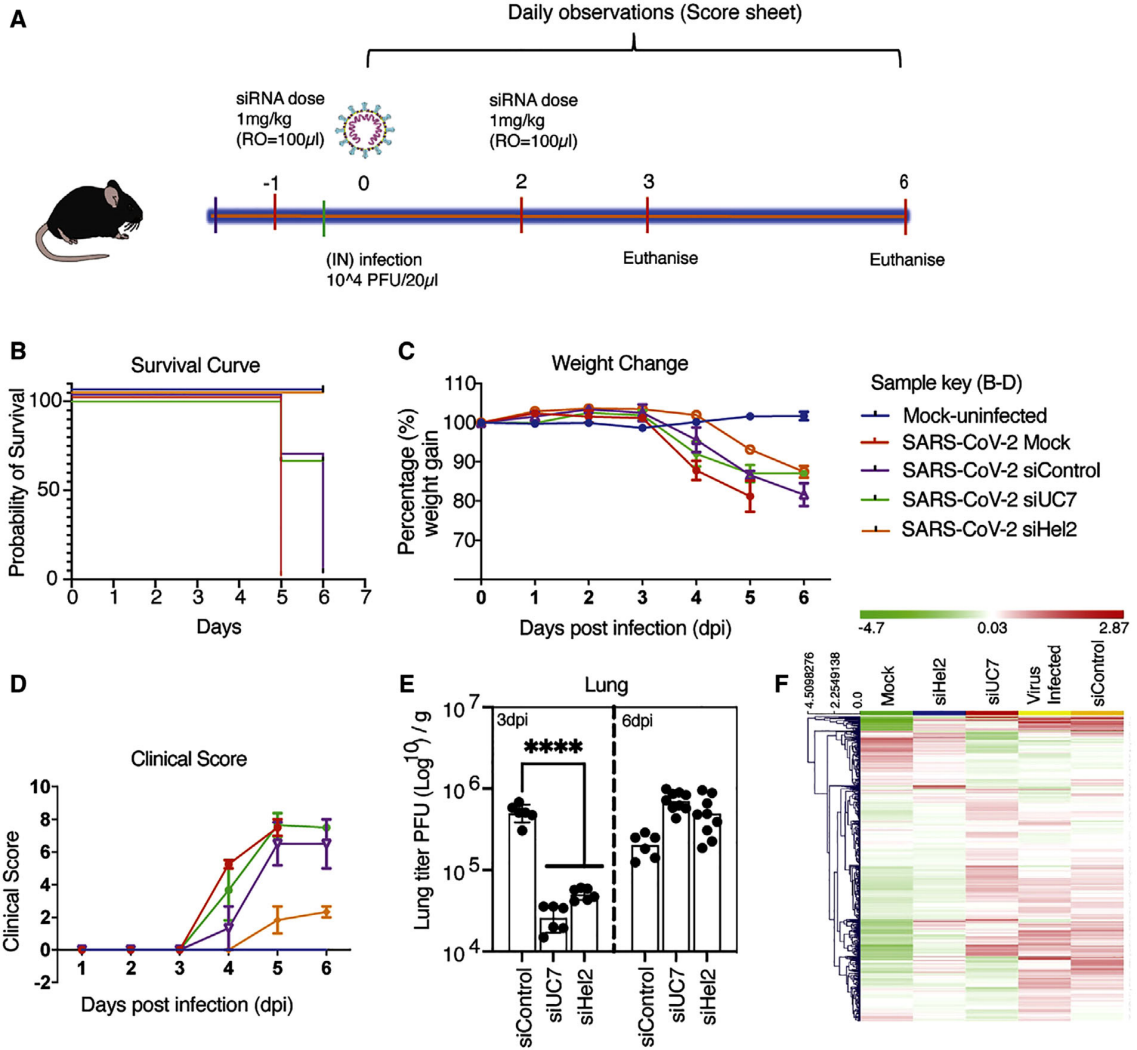
Systemic administration of siRNA results in suppression of COVID‐19. (A) Schematic of experimental time course and dose of siRNA therapeutic administered. (B) Kaplan Meier curve demonstrating survival probability, days 1–7. (C) Changes in weight measured over the experimental period. (D) Clinical score evaluated on movement, behaviour, and overall appearance. (E) Concentration of virions identified in lung tissue at days 3 and 6 post inoculation. (F) Unsupervised hierarchical cluster heatmap of immune gene expression in the lungs at day 6. Reproduced with permission.^[^ ^94a^ ^]^ Copyright 2021, Cell Press

siRNA therapeutics possess an additional advantage in that they can be delivered as a cocktail, where different siRNA target sequences can be delivered simultaneously. Multi‐targeting siRNA approaches such as this are also proving to be a viable option for treating COVID‐19 infection. In one highly promising approach, Idris et al. recently identified, and designed siRNA for three highly conserved COVID‐19 mRNA sequences. siRNA was delivered alone or simultaneously to target mRNA for RdRp, Helicase (Hel), and 5′ untranslated region (5′ UTR).^[^ ^94a^ ^]^ K18‐hACE2 transgenic mice expressing the human ACE2 receptor were inoculated with the COVID‐19 virus. At days 1 and 2 post inoculation, the mice were treated with 1 mg/kg in 100 ml of siRNA packaged into hydration of a freeze‐dried matrix (HFDM) lipid nanoparticles, administered via retro‐orbital injection (Figure 6). Treatment with siRNA was demonstrated to provide a survival advantage by repressing COVID‐19 infection. However, this effect was found to be only short term due to the intrinsically transient silencing effect of siRNA which, as demonstrated here, generally lasts between 24 and 48 h. Notably, several important patent applications relating to siRNA design, synthesis methodology and delivery have also been issued in the aim of targeting COVID‐19. Patents include WO2021206917A1, a patent for a double stranded RNA targeting ACE2. WO2021195025A1, an antisense treatment for COVID‐19. CN112574960A a siRNA that efficiently cuts the COVID‐19 genome and helps effectively capture virions. WO2021236763A2, a double stranded siRNA with the potential to treat COVID‐19, SARS and MERS infections. KR102272800B1, an oligonucleotide designed to prevent proliferation of COVID related infections, including mutated versions. WO2021206917A1 filed for the synthesis and development of oligonucleotides with the aim of improving compound stability and cellular uptake. US2021246448A1, a patent for a targeted therapeutic delivery vehicle.

Although it is well established, that one of the main disadvantages to siRNA therapeutics is that they require a nanoparticle delivery vehicle to deliver the siRNA into diseased cells, when it comes to controlling immune responses, the need for a nanoparticle delivery vehicle becomes advantageous, because nanoparticle can be designed to be either immunogenic, promoting an immune response or be lowly immunogenic, limiting pro‐inflammatory mediators, depending on the desired response.^[^ ^101^ ^]^

We are yet to find a COVID‐19 siRNA antiviral in the clinic, and because siRNA can be carefully designed to limit off‐target toxicity, it is our opinion that the reason for this is because a suitable delivery vehicle has not been identified that meets the criteria for delivering antiviral siRNA to the lungs. In this respect, we keenly await the findings from the siR‐7‐EM/KK‐46 Phase I clinical trial. Finally, targeting the lungs can be improved by the way in which the nanoparticle‐siRNA is delivered. Most notably, via intranasal delivery, a method whereby nanoparticle‐siRNA is directed through the nasal passages and down towards the lungs.

Intranasal delivery of therapeutic siRNA may present as an effective approach to treating COVID‐19 and other viruses that attack respiratory function. When siRNA is delivered in this manner it is minimally invasive, and because the nasal route limits exposure to systemic blood flow and exposure is mostly confined to the lungs, intranasal delivery has the potential to be safer than intravenous delivery that otherwise promotes siRNA delivery to major organs including the liver, heart, kidney, and spleen.^[^ ^102^ ^]^ Nevertheless, siRNA delivered intravenously is still prone to rapid mucociliary clearance, alveolar macrophage clearance and degradation by RNAses.^[^ ^103^ ^]^ In this respect, the nanoparticle vehicle carrying the siRNA is instrumental in avoiding clearance and preventing degradation.^[^ ^104^ ^]^ Because delivering siRNA intranasally preferentially targets lung tissue, the chance of the siRNA reaching target cells is significantly increased and the gene silencing effect potentially takes place more rapidly, suggesting that dosing can be kept relatively lower than if the siRNA was being delivered systemically.^[^^]^ The ability to deliver low dosages subsequently reduces the chance of off‐target toxicity and adverse immune reactions providing that both the siRNA sequence and carrier nanoparticles are carefully chosen and proven not to be immunogenic. The first siRNA antiviral for intranasal delivery of an siRNA (ALN‐RSV01) was targeted against RSV infection.^[^ ^105^ ^]^ RSV, like COVID‐19 is a single‐stranded RNA virus and belongs to the Paramyxoviridae family that also includes mumps, measles, rabies, Ebola.^[^ ^106^ ^]^ ALN‐RSV01 is designed by Alnylam, the company that successfully produced the first approved siRNA therapeutic, patisiran. ALN‐RSV01 was effective in early clinical trials and finished Phase II in 2018 (NCT00658086).^[^ ^107^ ^]^ However, results are yet to be published by Alnylam for this study. The ability to target RSV using the intranasal delivery route, suggests that siRNA can be administered in this way to target COVID‐19. For instance, Alnylam is currently undertaking pre‐clinical testing for intranasal delivery of 350 siRNA, targeted against COVID‐19. Whilst Sirnaomics are evaluating a COVID‐19 siRNA formulation designed to be administered using a easy to operate nebuliser.^[^ ^108^ ^]^

In summary, siRNA viral targeting strategies present as a promising way to shut down COVID‐19 viral genes in a safe non‐immunogenic way. This important aspect could potentially help to ensure that the immune system does not become overwhelmed if siRNA is used to complement other vaccine strategies that work to initiate an immune response. Together with this, the rapid and relatively straightforward synthesis of siRNA, and the ability to upscale production, may also prove extremely advantageous to the next generation COVIDs and other respiratory viruses that will in doubt follow.

## 4 CONCLUSION

The warning shots of the deadly SARS and MERS outbreaks of 2003 and 2012 respectively were, up until COVID‐19, shamefully ignored by governments and the scientific community alike. A plethora of reviews was written to warn and urge us to prepare.^[^ ^109^ ^]^ Even the well‐founded suggestions of a prominent virologist, that we begin to stockpile a collection of different antivirals, thereby preparing for future possible pathogenic outbreaks after the SARS breakout, went largely unnoticed.^[^ ^110^ ^]^ For this level of apathy, the world has paid a heavy emotional and financial price, of which, we will in doubt, all bear the brunt for years to come. Moving forward, and with the knowledge of hindsight, we must continue to strive to explore a range of different antiviral strategies that are suitable for every patient, regardless of their underlying health issues. This is vital, and something all newly approved drugs have been unable to promise. For this ‘every patient matters’ strategy to happen, a greater understanding of patient susceptibility to COVID‐19 is warranted so that it is possible to identify all potential mechanisms involved in pathogenicity and stringently monitor and rapidly report changes to the viral genome. siRNA antivirals may have the potential to switch off already identified gene influencers of infection and be adapted quickly in response to newly identified gene modifications brought about by virus mutations. Encouragingly, siRNAs for well‐chosen targets in various divergent pathologies have already received FDA approval after clinical trials found them to be highly effective and lowly toxic.^[^ ^80^, ^111^ ^]^ Because siRNA can be designed to target gene mutations with high specificity, siRNA antivirals may form a piece of the puzzle that provides one of the best ongoing solution to the mutational plasticity of the COVID's. However, for even the most promising siRNA antivirals in pre‐clinical studies, some work still remains to be done before they reach patients. Furthermore, it is unrealistic to proclaim that siRNA antivirals alone will provide the complete solution to this current crisis. Ultimately, what's fundamentally important moving forward is that the drive to find diverse, potent, cost‐effective remedies for this deadly infection must not wane, nor must the stockpiling of antivirals idea fade, because let's face it, if the COVID's have been steadily worsening in their ability to critically infect us since the 1960s, it is doubtful they will be stopping any time soon.^[^ ^7^ ^]^

## CONFLICT OF INTEREST

Thomas P. Davis is a member of the *Exploration* editorial board. The authors declare no conflict of interest.
